# A rare case report of recurrent atypical meningioma with multiple metastases treated with anti-PD-1 and anti-VEGF therapy

**DOI:** 10.1186/s12883-022-02919-4

**Published:** 2022-10-22

**Authors:** Jia-Li Zhao, Jing Liu, Ming Fang, Chen Luo, Zhen-Bang Gu, Long Huang

**Affiliations:** 1 Department of Oncology, The Second Affiliated Hospital of Nanchang University, 1 Minde Road, Nanchang, Jiangxi China; 2 JiangXi Key Laboratory of Clinical and Translational Cancer Research, Nanchang, China; 3 Department of Pathology, The Second Affiliated Hospital of Nanchang University, Nanchang, China; 4 Yangxin People’s Hospital of Hubei Province, Huangshi, China; 5 Medical School of Nanchang University, Nanchang, China

**Keywords:** Case report, Meningioma, Anti-PD-1, Anti-VEGF, Combined therapy

## Abstract

**Background:**

Meningioma is the most common type of primary intracranial tumor with 0.1–1% of all primary meningiomas have been reported to develop into metastases. However, there is no proven therapeutic strategy for multiple metastases of meningiomas.

**Case presentation:**

A 60-year-old female accepted total tumor resection of a right frontal lobe meningioma in September 2018, In October 2021, the patient was admitted to hospital because of cough and shortness of breath and diagnosed with metastatic meningiomas. The computed tomography (CT) scan revealed the presence of large masses in the right thoracic and abdominal cavity. After two cycles of anti-PD-1 and anti-VEGF treatment, the symptoms were relieved and the tumor was necrotic. Follow up to June 21, 2022, the patient has been given eleven cycles of the treatment every 3 weeks without tumor progression.

**Conclusions:**

This case showed combined anti-PD-1 and anti-VEGF treatment stimulates peripheral blood immune cells to kill metastatic meningioma cells. Whether combined immunotherapy is more effective for metastatic meningioma needs further exploration.

## Background

Meningioma is the most common type of primary intracranial tumor that is characterized by slow growth and good prognosis. However, meningiomas can be aggressive or even undergo malignant transformation in a small number of cases: 0.1–1% of all primary meningiomas have been reported to develop into metastases [1–3]. However, there is no proven therapeutic strategy for multiple metastases of meningiomas.

## Case presentation

**Fig. 1:**
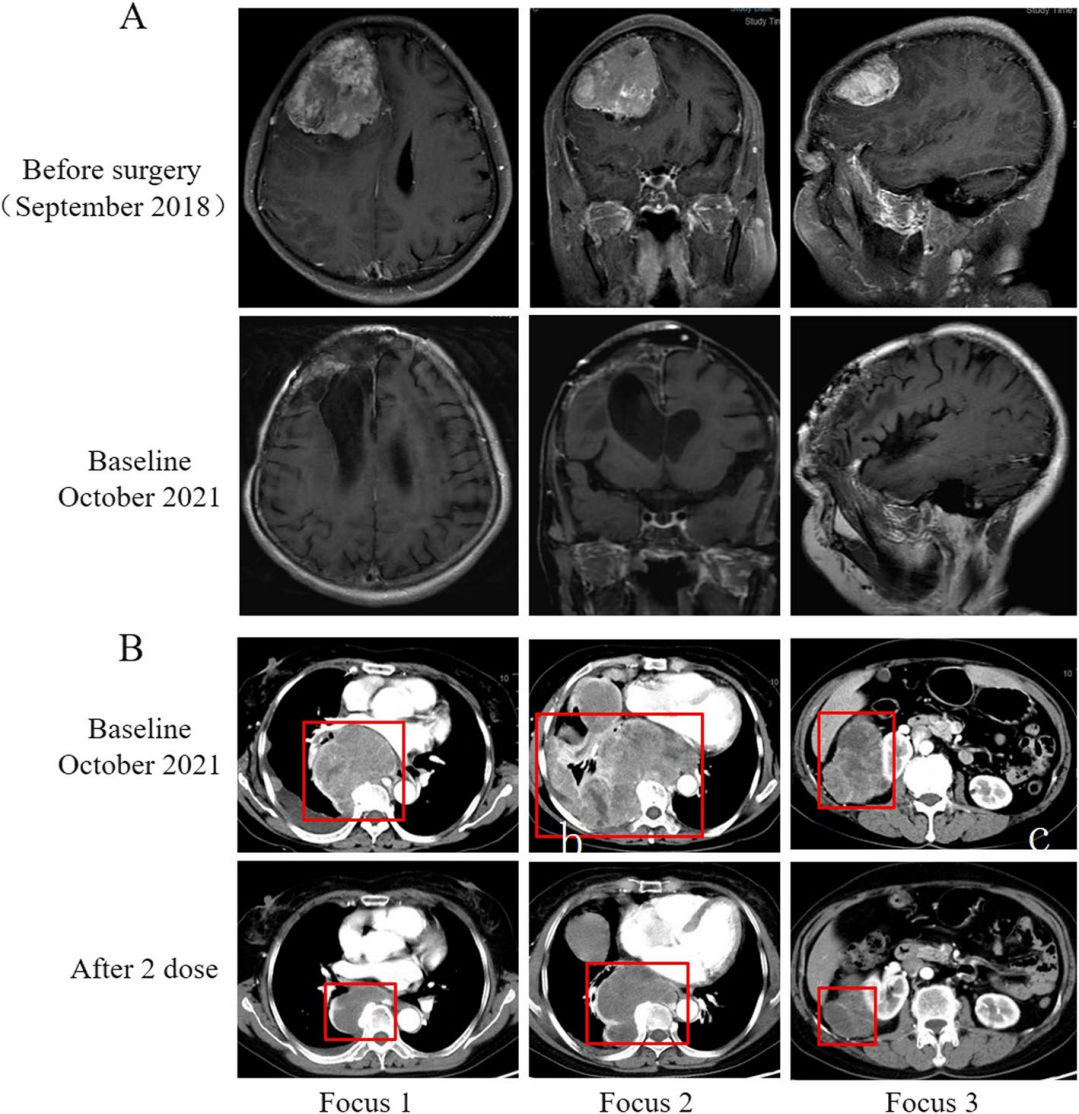
**A** Magnetic resonance imaging (MRI) of the primary meningioma. MRI scans showed that the primary meningioma was located in the right frontal lobe of the convexity of the brain and could be completely resected. **B** Computed tomography (CT) revealed significant shrinkage of the tumor after two cycles of treatment compared with the size of the tumor before treatment

**Fig. 2:**
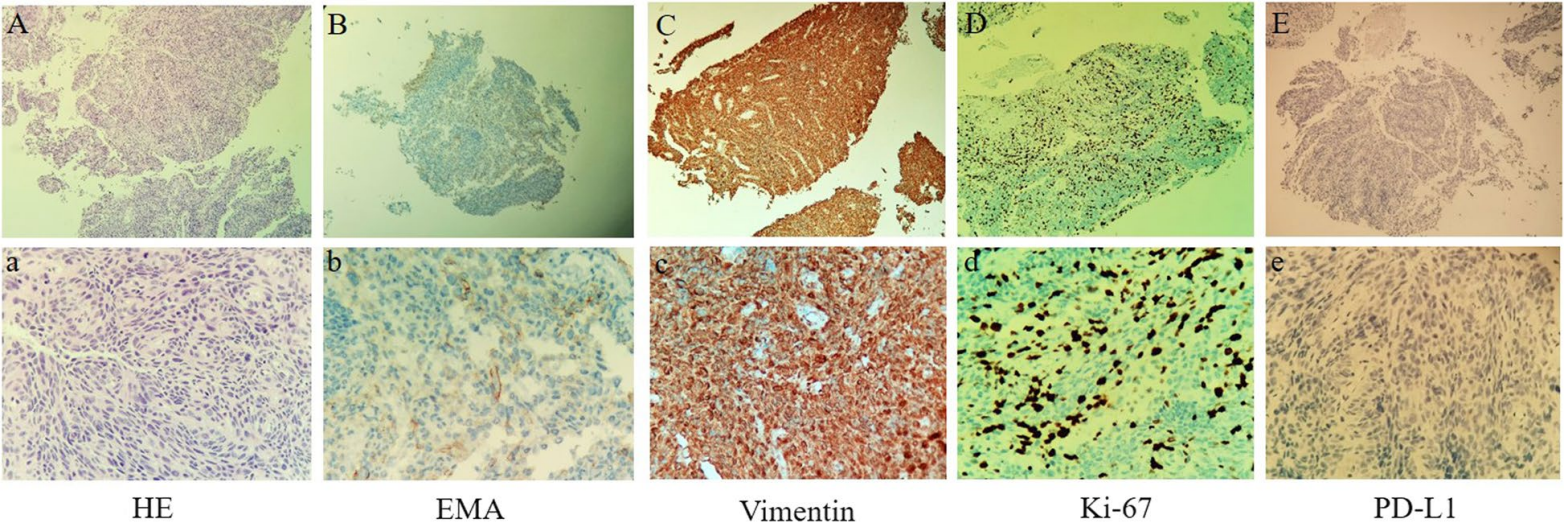
Morphologic features and IHC findings of the biopsy samples. Hematoxylin and eosin staining (HE) revealed fusiform cancer cells (A, × 100; a, × 400). IHC revealed that the cancer cells were positive for EMA, Vimentin, PD-L1, and Ki-67 (B–D, × 100; b–d, × 400) and negative for PD-L1 (E, × 100; e, × 400)

**Fig. 3:**
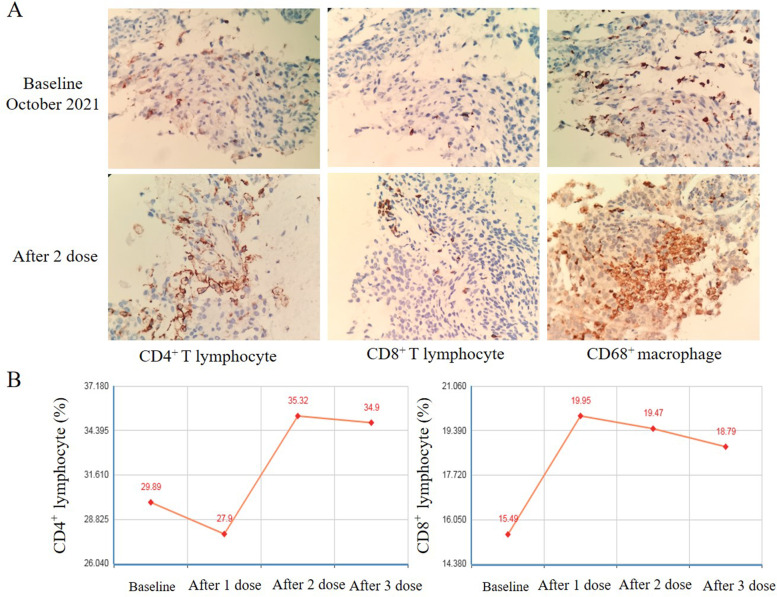
**A** Immunohistochemistry (IHC) revealed a significant increase in infiltration of CD4^+^ T lymphocyte, CD8^+^ T lymphocyte, and CD68^+^ macrophage in the tumor microenvironment compared with that before treatment (× 400). **B** The numbers of CD4^+^ T lymphocyte and CD8^+^ T lymphocyte also continued to increase as he tumor shrank in the peripheral blood

A 60-year-old female underwent total tumor resection of a right frontal lobe meningioma which located on the convexity of the brain in September 2018, and the pathological diagnosis was atypical meningioma (Fig. 1A). In October 2021, the patient was admitted to the hospital with cough and shortness of breath. A computed tomography (CT) scan revealed the presence of large masses in the right thoracic and abdominal cavity and no recurrence was found in craniocerebral magnetic resonance imaging (MRI) (Fig. 1A). The histological features of the lung tumor were similar to that of the brain mass, and the tumor cells were positive for vimentin, EMA, and Ki-67, and negative for TTF1, PD-L1, P40, and chromogranin (Fig. 2). Based on these biopsy and immunohistochemistry (IHC) findings, the masses were identified as metastatic meningiomas. The patient was treated with the anti-PD-1 agent camrelizumab (200 mg, Day 1) combined with the anti-VEGF agent anlotinib (10 mg, Days 1–14) every 3 weeks without radiation treatment. After two cycles of this regimen, the patient’s symptoms were completely resolved without no other adverse events, and CT revealed that the tumor had shrunk significantly by > 80% (Fig. 1B). Biopsy performed after the two cycles of treatment and immunohistochemistry analysis revealed that infiltration of CD4^+^ T lymphocyte, CD8^+^ T lymphocyte, and CD68^+^ macrophage in the tumor microenvironment was significantly increased compared with that before treatment (Fig. 3A). The number of peripheral blood CD4^+^T lymphocyte and CD8^+^T lymphocyte continued to increase as the tumor shrank (Fig. 3B). These findings indicate that combined anti-PD-1 and anti-VEGF treatment stimulates peripheral blood immune cells to kill metastatic meningioma cells. The patient has been given eleven cycles of the treatment every 3 weeks from October 21, 2021 to June 21, 2022 without tumor progression.

## Discussion and conclusions

Meningiomas are highly vascularized tumors [4]. The VEGF-A-driven system of tumor angiogenesis is still a target for adjuvant therapy in malignant recurrent meningioma disease [5]. Bevacizumab, a monoclonal antibody that targets vascular endothelial growth factor (VEGF),was demonstrated to prolong disease stabilization in two phase II prospective studies of bevacizumab in meningioma [6, 7]. Additionally, immunotherapy has shown clinical benefits in some advanced malignancies of the central nervous system [8], but a phase 2 clinical trial on treatment of recurrent grade 2/3 meningioma with nivolumab, an anti-programmed cell death-1(anti-PD-1) treatment, showed that it did not result in an increase in six-month progression-free (PFS-6) survival [9]. However, another latest phase 2 clinical trail on treatment of recurrent and residual high-grade meningiomas with pembrolizumab(a PD-1 inhibitor), it did met its primary endpoint of PFS-6 rate [10]. The promising results associated with the use of anti-PD-1 has led to increased interest in using concurrent anti-PD-1 and anti-VEGF therapy for multiple metastases of meningiomas.

Combining anti–PD-1 and anti-VEGF therapies has shown synergy and positive outcomes in phases I to III studies and appear to be particularly effective in the setting of high levels of VEGF [11]. Herein, we report the first case of a patient with multiple metastases of meningiomas who was successfully treated with anti-PD-1 and anti-VEGF therapeutic regimen without radiation treatment. Although it’s difficult to exclude the possible synergy contributions of combined treatment regimen to the favorable response. The outcomes in the present case warrant further clinical trials on concurrent anti-PD-1 plus anti-VEGF therapy for the treatment of recurrent distant metastasis of meningiomas.

## Data Availability

The datasets used during the current study are available from the corresponding author on reasonable request.

## Abbreviations

CT: Computed tomography
MRI: Magnetic resonance imaging
IHC: Immunohistochemistry
anti-PD-1: Anti-programmed cell death-1
VEGF: Vascular endothelial growth factor
PFS-6: Six-month progression-free
TPS: Tumor Proportion Score
CPS: Combined Positive Score
